# Bilingual language control during single-language production: does relocation to a new linguistic environment change it?

**DOI:** 10.1098/rsos.241071

**Published:** 2025-02-19

**Authors:** Angela de Bruin, Cong Liu, Danijela Trenkic, Marion Coumel

**Affiliations:** ^1^ Department of Psychology, University of York, York YO10 5DD, UK; ^2^ Department of Psychology, Qingdao University, Qingdao, People’s Republic of China; ^3^ Department of Education, University of York, York, UK

**Keywords:** bilingualism, proactive language control, language environment, language production

## Abstract

A bilingual’s two languages are simultaneously active and competing for selection, even when only one language is used. To manage this competition, bilinguals apply language control. We examined how bilinguals apply control across two single-language tasks and how this language control might adapt to the language environment bilinguals live in. We conducted a longitudinal study with Mandarin–English bilinguals who moved from China to the UK and a control group staying in China. Participants completed a picture-naming task and a verbal-fluency task twice, approximately seven months apart. We examined language order effects by comparing performance in each language when it was used first versus after the other language. While the L2 benefited from being used second, L1 performance benefited less or even deteriorated after L2 use. This suggests bilinguals proactively applied language control, especially during L2 use, to manage the anticipated language competition from the L1. However, these effects did not change after relocation to the UK, nor did they differ between the groups. This suggests that while language control is a core part of language production, the language environment a bilingual lives in might not have a defining impact on the exact way this language control is applied.

## 1. Introduction

Even though bilinguals speak multiple languages, they frequently encounter contexts in which only one of those languages can be used. For instance, a Mandarin–English bilingual can only use English when having a conversation with an English-speaking monolingual. Still, even in these single-language contexts, the other language (Mandarin in this example) is active and continues to compete for selection. Evidence for this so-called non-selective language activation has been observed during both language comprehension (e.g. [1]) and production (e.g. [2]).

To manage ongoing language co-activation and competition, bilinguals use language control. This language control can include activation of the target language (e.g. activating words in English) but has also been linked to inhibition of the language not currently in use to manage interference (e.g. suppressing Mandarin words [3]). Inhibition has been argued to affect bilingual production when the previously inhibited language has to be used again as the target language at a later point, requiring re-activation of that language. In single-language contexts where the bilingual knows that only one language can be used, this control can be applied proactively. That is, bilinguals could apply these language-control mechanisms in advance and in anticipation of potential interference from the language they cannot use in that context. This language control might be applied relative to the strength of each language. If a bilingual has a dominant language (here referred to as the first language, L1) that they are more proficient in and use more often, it might take more language control to avoid interference from it when using a less-dominant second language (L2), compared to the control needed to avoid L2 interference during L1 production. Bilinguals have therefore been argued to apply more L1 control during L2 production than vice versa (e.g. [3,4]).

However, bilinguals differ in how much time they spend in single-language contexts in their daily lives, and thus potentially in the degree and manner in which they use their language control. Following the adaptive control hypothesis [5], the language environment(s) a bilingual communicates in might shape their language-control mechanisms. Bilinguals mostly living in L1 single-language contexts do not necessarily have to apply language control very strongly, in particular if their L2 has a lower proficiency level. In contrast, bilinguals spending more time in L2 single-language contexts might develop their (proactive) language control differently to accommodate to these language contexts. If this is the case, proactive language control should differ between these two types of bilinguals, depending on the environment they live in. However, bilinguals do not always stay in the same language environment throughout their lives, and their daily language environment might change substantially as a result of moving to a new city or a new country.

In the current study, we therefore examined if and how proactive language control adapts to bilinguals changing their daily-life language environment. We tested Mandarin-dominant Mandarin–English bilinguals soon after moving from China to the UK and seven months later. Participants acquired Mandarin from birth, were more proficient in Mandarin than English, and were predominantly using Mandarin before starting the study (i.e. they were living in an L1-dominant environment). They then moved to an environment that required more frequent use of their L2. We compared these bilinguals to a control group staying in China. Changes in their proactive language control would support the hypothesis that language control is adaptive to a bilingual’s language environment. We furthermore examined language control in two tasks (picture naming and verbal fluency) varying in their lexical selection demands, to examine whether (a potential change in) language control is task dependent.

### 1.1. Proactive language control in single-language contexts

During language production, bilinguals have been argued to use language control reactively as well as proactively. Reactive control is described as being applied in response to conflict or competition, for example at the moment when bilinguals have to switch languages. Proactive control can be described as being more sustained than reactive control and can be applied in anticipation of upcoming competition (see [6] for a review). Proactive control can be studied through various types of tasks, including comparisons between single- and dual-language contexts (see [6]). However, comparisons between those two contexts typically focus on how proactive control is used in *dual-language* contexts when two languages are used interchangeably. Focusing on single-language contexts only, proactive control has been studied in the form of language order effects (e.g. [4]). In this type of studies, participants are typically asked to name pictures in L1-only or L2-only blocks, while manipulating the order in which they do these single-language blocks (L1 first or after L2). The main finding from these studies, as reviewed in more detail below, is that language order effects differ for the L1 versus L2. Typically, L1 performance is found to be poorer when the L1 is used after the L2 (compared to when the L1 is used first; e.g. [7]) or L1 performance benefits less from task repetition (i.e. from doing the same task again, but in another language) than the L2 (e.g. [4]). These patterns are typically interpreted as providing evidence for language control being needed in particular while using the L2, potentially in the form of L1 inhibition when using the L2 (e.g. [4,6]). The exact patterns, however, might vary depending on the stimulus presentation (e.g. repeating the same pictures across languages or not), the task used (picture naming or verbal fluency) and the type of bilinguals tested. These factors were further examined in the current study and are introduced in more detail below.

Misra *et al*. [4] showed different L1 and L2 language order effects in a picture-naming task with Chinese–English bilinguals. In the L2, pictures were named significantly faster when the L2 block followed the L1 block than the other way around. Given that the same pictures were used in both the L1 and the L2 blocks, this facilitation is likely the consequence of picture repetition and task practice. However, this facilitation was not observed for the L1, which did not benefit from participants having named the same pictures in the L2 first. These effects were observed in terms of naming times and did not influence accuracy. In addition to behavioural performance, event-related potentials (ERPs) were measured. The L1 and L2 order effects showed different directions in a negative going wave with the time course of an N2 component, which is often interpreted to reflect top-down control. Together with the behavioural data, this suggests bilinguals applied L1 control during L2 naming, which reduced L1 lexical access and resulted in bilinguals needing relatively more time to re-activate the L1 when it had to be used again next.

In their picture-naming task, Misra *et al*. [4] repeated the same pictures in both language blocks. Therefore, the observed patterns of language control could have been item-specific. For instance, when naming the picture of a horse in Mandarin, a bilingual might only (reactively) suppress the word ‘horse’ in English but not other English words. Instead of, or in addition to it, bilinguals could also apply language control globally across all words in a language, regardless of the item to be named. To study whether the control applied is global and potentially more proactive, other studies have included both repeated and new pictures when studying language order effects. Branzi *et al*. [7] tested highly proficient Catalan–Spanish participants. They named pictures in three blocks. The first block and third block were named in the same language; the second block was named in the other language (i.e. L1–L2–L1 or L2–L1–L2). The second block included repeated pictures already named in the first block and new pictures. Starting with the comparison between the first and second blocks, repeated pictures showed an order effect, with faster L2 naming after L1 naming (L2 facilitation) but no L1 facilitation. These findings are similar to those of Misra *et al*. [4]. For unrepeated (new) pictures, the L2 showed no order effect anymore. The L1 now showed poorer performance when it was used after the L2. This suggests that when the facilitation of picture repetition is removed, L1 words are less accessible after L2 use than before L2 use. This furthermore suggests that language control can be applied proactively and globally across all words, as control over translation equivalents only should not have affected the new (unrepeated) picture names (see also [8], for evidence supporting both lexicon-global and item-specific language control). The comparison of language order effects between the first and second blocks was done between participants. This study also included a third block to compare, within-participant, the difference between the first block (L1 naming baseline) and the third block (L1 naming after L2 naming). Only considering new items, L1 naming was again found to be significantly worse after L2 naming than in the baseline first block. In contrast, L2 performance did not differ for the first versus third block. This again suggests the L1 and L2 differ in proactive language control. Furthermore, studies have shown (e.g. [4,9]) that these language order effects continue to influence production for at least a few minutes (i.e. they do not disappear immediately after the start of the new language context), strongly suggesting these effects reflect sustained and proactive language control.

While these L1/L2 order effects are often linked to inhibition, this is not the only interpretation offered. While the ERP data in Misra *et al*. [4] mostly showed changes in components previously associated with inhibition, this was not the case in Branzi *et al*.’s [7] ERP data, which observed effects in the P2 rather than the N2. The P2 is not usually associated with inhibition. Indeed, language order effects differing between languages are often explained through L1 inhibition, but they can also be explained through L2 over-activation. If bilinguals proactively over-activate the L2 (to allow for easier L2 use), this L2 over-activation might persist when using the L1 in the next block and this could explain P2 effects and reduced L1 performance. Branzi *et al*. [7] argue that these language-control mechanisms might furthermore differ depending on the bilingual groups tested (e.g. highly proficient Catalan–Spanish bilinguals in Branzi’s study versus unbalanced Chinese–English bilinguals in Misra’s study). Thus, it is possible that the involvement of L1 inhibition or L2 over-activation differs depending on the bilingual’s language profile.

Further research examining the nature of proactive control in different participant groups is provided by Van Assche *et al*. [10], who tested Dutch–English and Chinese–English bilinguals on a verbal-fluency task. Participants were asked to name words starting with a specific letter or phoneme in each language. The second block in the other language used either the same letter/phoneme or a different one. Both language groups showed a language order effect that differed between the L1 and L2 when the letter/phoneme was repeated. L1 performance was worse when the L1 was used second (after the L2 block) than when it was used first. In contrast, L2 performance did not differ between being used first or second. While their study focused on the verbal-fluency tasks, it also included a short picture-naming task as a proficiency measure. The language order was counterbalanced within that task too and therefore also examined. Contrary to the verbal-fluency task, however, the picture-naming task did not show any order effects. This suggested that, in some cases, the verbal-fluency task might be more sensitive to language-control effects.

Furthermore, different patterns were observed for Chinese–English and Dutch–English bilinguals in terms of lexicon-global control. When different letters/phonemes were used, only the Chinese–English bilinguals showed different L1–L2 language order effects. This suggests that while various bilingual groups apply local control, lexicon-global control across all words in a language can vary depending on the type of bilingual group tested. In this specific study, Chinese–English bilinguals (who showed global and local control) had been living in an L2-dominant environment, while Dutch–English bilinguals (who showed local control only) were living in an L1-dominant environment. In the presence of large differences between the languages (e.g. Chinese and English are far more dissimilar than Dutch and English in terms of orthography, phonology, lexicon and syntax), any group differences might be due to variables other than language environment. However, a recent study [11] provides further evidence that individual differences exist in language control in these types of contexts. They examined the language order effect (reported in [9]) relative to a bilingual’s L1 activation (i.e. baseline L1 naming times) and L2 activation (baseline L2 naming times). The language order effect (slower L1 naming after L2 use) was largest for unbalanced bilinguals, who showed a larger difference between their L1 and L2 activation. This suggests these bilinguals in particular apply language control during L2 use, either through more L1 inhibition and/or through more L2 over-activation. Together, these two studies [10,11] suggest language control can depend on the bilingual’s language background.

### 1.2. Language control adaptation

Thus, there is strong evidence that bilinguals use language control in single-language contexts, with some further suggestions that proactive language control in single-language contexts might differ between bilinguals. Indeed, the adaptive control hypothesis [5] argues that different bilinguals, depending on the language environment they communicate in, might use their language control mechanisms differently. They describe single-language contexts as mostly relying on goal maintenance (keeping the goal in mind of using one specific language) and interference control (monitoring conflict from the other language and suppressing interference). However, how exactly these mechanisms are shaped and used could depend on a bilingual’s language overall daily life environment. A further distinction can be made between time spent in L1- versus L2-dominant contexts. L1 contexts might require relatively little language control for unbalanced bilinguals (whose L1 is more dominant than the L2), as the most dominant language is used most of the time. In contrast, L2 contexts might require more language control over the L1 for such unbalanced bilinguals. Spending more time in L2 contexts could shape language control mechanisms to allow for more optimal communication. For example, bilinguals who spend more time in L2 contexts could continuously apply more L1 inhibition (or L2 over-activation) to allow for efficient L2 communication without L1 interference, compared to bilinguals spending most time in L1 contexts. This would align with group differences observed in Van Assche *et al*. [10]. Alternatively, bilinguals who spend more time in L2 contexts can become more balanced in their languages’ activation levels and, in line with Casado *et al*. [11], could show smaller language control effects, potentially because they require less L1 control and/or use their language control more efficiently.

Despite language experiences being hypothesized to play an important role in and potentially shape language-control mechanisms, research on this topic is scarce. Most previous research has either focused on *non-linguistic executive* control (e.g. [12–15]) and language-switching training (e.g. [16]) or assessed group differences in terms of language production without directly testing language control mechanisms (e.g. [17,18]). For instance, Beatty-Martínez *et al*. [17] compared three different groups of Spanish–English bilinguals living in different language environments (Spain, Puerto Rico and Pennsylvania). Despite speaking the same languages, these groups varied in their performance on both a verbal fluency and a picture-naming task. This study thus supports the argument that bilingual groups can vary in their language production, depending on the environment they are living in. However, although some differences were also observed in terms of executive control, the language production components of this study focused on lexical accessibility (e.g. how many words could be retrieved or how fast they were retrieved) and did not manipulate language control.

Linck *et al.* [18] also found differences in language production between different groups of English–Spanish speakers and attributed those differences to language control specifically. One participant group had moved to Spain (immersion group, tested after three months abroad), while a classroom group stayed in the USA and took part in a Spanish course there. Participants completed a semantic verbal-fluency task (in addition to a translation recognition task). Participants produced more exemplars in their L1 English than in L2 Spanish. This language difference, however, was smaller for the immersion group, which produced more L2 words and fewer L1 words than the classroom group. These findings suggest that lexical access in the L1 was reduced in the immersion group. These findings align with the language attrition literature showing changes in the L1 in relation to an increase in L2 use or development (see [19]). Linck *et al.* [18] explain their findings through L2 immersion affecting language competition, and the language control bilinguals might use to manage this competition, with a focus on L1 inhibition. Participants in the L2 immersion group could have inhibited their L1 while being immersed, to allow for easier L2 access. This in turn could lead to reduced L1 access (see also [20]).

However, in the absence of a language competition or control manipulation specifically, the L1 changes observed in the verbal-fluency task in Linck *et al*. [18] could also be explained through a reduction of L1 use in the immersion group. This could slow down or hinder L1 retrieval without a change in language control. Indeed, the weaker links hypothesis (e.g. [21]) proposes that less frequent use of a language could weaken the links between concepts and lexical forms in that language, leading to slower retrieval. Explanations focusing on language competition and control or reduced language (L1) use are not necessarily mutually exclusive (e.g. the amount of language use could modulate the amount of language competition and thus control needed [22]). However, the two accounts differ in the proposed underlying mechanisms, with one interpretation focusing on reduced lexical access due to reduced language use and the other explanation focusing on changes in language control. In the absence of a manipulation targeting language control, it is difficult to attribute the observed group differences to control specifically. Crucially, language order was also confounded between the two groups. The immersion group always completed the verbal-fluency task in their L2 first and L1 second, while the classroom group always followed the opposite language order. Thus, poorer L1 performance could be explained through the immersion group always completing the task in the L1 *after* the L2, which (as reviewed above) has been associated with poorer performance relative to the opposite language order. Furthermore, both Linck *et al*. [18] and Beatty-Martínez *et al*. [17] worked with between-group comparisons. Comparing groups could introduce several between-group differences beyond the language environment as such, including differences in language proficiency. Finally, when two groups are only tested once without a baseline measure, as done in these studies, it is difficult to attribute group differences to changes in language environment or increased L2 immersion specifically. Thus, the patterns observed in these studies cannot directly speak to effects of language environment (changes) on language control.

To overcome some of the issues associated with between-group comparisons, Baus *et al*. [23] assessed language production in German students at the start of their semester studying abroad in Spain and after four months (‘immersed group’). They were compared to a control group of Spanish monolinguals. To examine the potential role of language use and to assess the weaker links hypothesis (e.g. [21]), the study also manipulated word frequency. If any changes in lexical retrieval (poorer L1 production after L2 immersion) are related to less L1 use, lower frequency words should be affected more strongly. It is those low-frequency words in particular that participants use even less in the L1 while in an L2 context, while high-frequency words continue to be used relatively frequently in both languages. In contrast, if reduced L1 performance is related to increased L1 inhibition, these effects were hypothesized to apply to both low- and high-frequency words equally, or most strongly to high-frequency words. High-frequency words are expected to be used more in the L2. If their L1 translation equivalents are suppressed more, high-frequency words should show the largest changes after L2 immersion.

The immersed group showed no change with time in terms of the number of L1-German words produced in the verbal fluency task, but they did produce a relatively larger number of cognates (similar word form and meaning in their L1 and L2) after four months. Their L1-German picture-naming performance also showed slower naming after four months in Spain than at the beginning, in particular for low-frequency non-cognate words. These changes with time were not present in the control group (although it should be noted they completed this task in Spanish, their L1, rather than German). Overall, these results again show that L1 production can change after immersion in an L2 context. The finding that these changes concerned low-frequency (non-cognate) words, in particular, was more easily explained through changes in L1 use (weaker links hypothesis) than through changes in language competition or control. These findings align with Botezatu *et al*.’s [24] study, whose picture-naming task also showed poorer L1 performance for an immersed L2 group on low-frequency items in particular, again supporting the weaker links hypothesis. In contrast, while Casado *et al*. [25] suggested that re-immersion (back in the L1 environment) can also influence language production, this was found to affect high-frequency words the most. However, none of these studies manipulated or measured language control.

Thus, the literature on single-language production and language environment so far has shown that L1 production can differ depending on the language environment a bilingual lives in. Typically, L1 production is found to be slower/reduced for bilinguals immersed in an L2 environment than for bilinguals living in an L1 environment, although these results are not always observed in all tasks. However, studies so far have not specifically assessed proactive language control (e.g. through language order manipulations), leaving open different interpretations. While some studies focus on inhibition accounts, others have argued (mostly through manipulating frequency) that L1 changes or differences can more easily be explained through reduced L1 use leading to poorer or slower lexical access (in line with the weaker links hypothesis). More generally, studies so far have typically compared different groups of bilinguals, which introduces other between-group differences (e.g. proficiency differences) that can impact L1 production. Where bilinguals were followed throughout their first months in a new language environment [23], they were compared to a control group of *monolinguals* doing the task in a different language.

### 1.3. Current study

The current study therefore examined changes in L1 and L2 production longitudinally within a group of Mandarin–English bilinguals who moved from China to the UK (‘move group’). We will refer here to ‘Mandarin’ rather than ‘Chinese’ as we focused our recruitment on Mandarin speakers. While China is an L1-dominant environment for these bilinguals, the UK requires more L2 use. This can occur in L2 single-language contexts as well as in dual-language contexts, which have also been linked to proactive L1 control (e.g. [6]). Participants completed a series of background measures before they moved to the UK and then completed two sessions in the laboratory: once immediately after arrival in the UK (session A) and once approximately seven months later (session B). We also included a control group of Mandarin–English bilinguals who stayed in China throughout the study, to examine any test–retest effects in the tasks of interest.

While previous studies have focused on more general changes or differences in L1/L2 word retrieval (e.g. number of words produced in verbal-fluency tasks or overall reaction times (RTs) in picture-naming tasks), we focused on proactive language control. To do this, we manipulated the language order in which participants completed the single-language tasks (i.e. L1 before or after the L2). We furthermore compared two tasks (verbal fluency and picture naming) that vary in their demands on the retrieval of specific words. While picture-naming tasks require participants to produce specific words (high demands on lexical retrieval), verbal-fluency tasks allow for more freedom to retrieve lexical items as preferred, as long as they belong to a given semantic category. Language control could be argued to be more strongly needed in picture-naming tasks due to the higher demands on lexical retrieval, which in turn could place higher demands on L1 control during L2 naming. However, previous studies vary in the results observed for verbal-fluency versus picture-naming tasks. Baus *et al*. [23] indeed mostly observed changes in the picture-naming task, in line with a more control-demanding task perhaps being more sensitive to language-environment changes. In contrast, Van Assche *et al*. [10] observed different L1–L2 language order effects on verbal-fluency tasks but not during picture naming, suggesting the way proactive language control is applied might differ between the two. The current study therefore compared both tasks. Our study design also allowed us to examine control over specific words (translation equivalents in the picture-naming task, local control) as well as over other words in the language more globally (through the use of different semantic categories in the verbal-fluency task and unrepeated pictures in the picture-naming task). Finally, while language order is often manipulated between participants (with the exception of [7]), we also manipulated it in a within-participant design in the picture naming task.

Our main research question examined whether language control (both item-specific and lexicon-global) in different production tasks adapts to the language environment a bilingual lives in (adaptive control hypothesis [5]). We furthermore distinguished between potential changes in general language production performance due to lower L1/more L2 use (i.e. weaker links hypothesis) and actual changes in proactive language control. Our hypotheses were as follows. In line with previous literature (e.g. [18,23]), we expected changes in L1 and L2 production in the move group (*Hypothesis 1*). If those changes are specific to the change in language environment, they should be absent in the control group and arise as a significant interaction between group (move group versus control group) and session (A versus B). We furthermore expected these changes to interact with language, such that L1 performance would decrease, while L2 performance would improve (*Hypothesis 2*). Such general changes in L1 and L2 performance could purely be related to lower L1 use in the move group after moving, affecting ease of L1 retrieval (weaker links hypothesis).

*Hypothesis 3* focused on changes in language control, the main aim of our study. We expected an interaction between language order (whether a given language was used first or second) and language (L1 or L2), with L1 performance benefiting less than the L2 from being used second, or even suffering as a result of it. Importantly, it is the interaction between language order and language that would reflect language control, with the language order effects going in opposite directions for the L1 and L2, or (in the case of task repetition facilitation for both) being smaller for the L1 than L2. If proactive control does not change with a change in language environment, overall L1/L2 performance might change between sessions in the move group, but language order effects (in interaction with language) should not interact with session. However, if proactive control changes in the move group over time, we expected an interaction between session, language and language order. Two possible directions were hypothesized. If the move to an L2 environment resulted in bilinguals applying more L1 inhibition, we expected a larger, more negative, L1 language order effect in session B (in line with Linck *et al*.’s [18] argument). Alternatively, if a change in language environment results in more efficient language control, we expected more optimal language control and therefore smaller language order × language effects in session B.

We focused on changes within the move group as a whole (relative to the control group). In addition, we also considered that there might be individual differences in daily language experiences within the move group, in particular concerning how often they used the L1 and L2 while in the UK. This in turn could relate to language control (cf. [11]). As a final question (*Hypothesis 4*), we therefore examined a potential relationship between individual differences in daily-life language profile (age of acquisition, language proficiency, language use and time spent in L1 and L2 single-language contexts) and performance in the verbal-fluency and picture-naming tasks. We again examined this question in relation to (i) overall lexical access (i.e. overall L1 and L2 performance) and (ii) language control (in the form of language order × language effects). If these language-profile predictors related to lexical access more generally, we expected an interaction with language in terms of overall performance (e.g. participants with a higher L2 proficiency showing better L2 performance). However, if these language-profile predictors are related to language control, we hypothesized the daily-life language experiences to interact with language and language order (e.g. smaller language order × language effects for bilinguals who spend more time in L2 single-language contexts). We examined these relationships in session B as well as in relation to changes between the two sessions.

## 2. Methods

The pre-registrations, stimuli, datasets and analysis scripts are available on the Open Science Framework (OSF; https://osf.io/a24xv/). We created two separate pre-registrations (available in the ‘Registrations’ section of the OSF page): one focusing on the changes in the move versus control group and one focusing on the individual-difference analyses within the move group. Both pre-registrations also address the dual-language tasks included in the project, which are reported in a separate paper that can also be found on the OSF page [26]. Our pre-registration also included an additional hypothesis regarding the role of picture frequency (comparing naming of low- and high-frequency pictures). Given the lower sample size than planned, we deviated from the pre-registration by not including frequency in the analyses presented in the main paper. However, the analyses including frequency are reported in the electronic supplementary material.

### 2.1. Participants

The study was completed by two groups of Mandarin–English bilinguals. Of main interest was the group who moved from China (Mandarin-dominant environment) to the UK (English-dominant environment). We compared these participants (‘move group’) to a control group who stayed in China. All participants were tested in two waves across two academic years (2021−2022 and 2022−2023). The move group participants completed parts of the study before they moved (baseline assessment), shortly after arriving in the UK (session A) and after approximately seven months in the UK (session B). The control group completed the same tasks following a similar timeline. All participants had normal or corrected-to-normal vision and no known neurological, reading or hearing impairments. The study was approved by the Ethics Committee of the Department of Psychology at the University of York (approval number 961, with additional approval for control-group testing from the Department of Psychology at Qingdao University). Participants provided informed consent before each part of the study.

The study was completed by 114 participants (53 in the move group and 61 in the control group). An additional 44 participants started the study but either did not continue after the online baseline assessment (*n* = 23) or after session A (*n* = 21). The original study plans and power analyses (based on simulations using *simR*) aimed to start with 200 participants completing the baseline assessment. This would have given us over 95% power to detect medium-sized effects in relation to changes in language control between sessions (interaction language × language order × session) in the move group. Unfortunately, the sample size was lower than planned as a consequence of fewer people signing up and a higher drop-out rate than expected (28% relative to the first baseline assessment). Both factors were largely related to the study being completed during the COVID-19 pandemic. This greatly reduced the number of participants who could move from China to the UK to pursue an academic degree abroad, and thus both the number of participants who signed up initially and the number of people who actually moved to the UK after completing the baseline assessment. We therefore also conducted further analyses using *simR* to understand how much power the final sample size provided for the three-way interaction between language, language order and session in the move group (i.e. a change in language control in the group who changed their language environment) as well as the four-way interaction with group. To do this, we simulated models based on the models reported in §3 but changing the RTs to reflect small-to-medium effect sizes (cf. [27]). We then compared these simulated models to simulations without the effects of interest (without the interaction), with power analyses using *simR* indicating how often the simulated models with and without the effect of interest differed significantly using the number of participants included in the real analyses. We did this for a range of RTs reflecting different effect sizes. In both tasks, power was still over 70% to detect small-to-medium-sized changes in language control between sessions.

The move and control groups were comparable in terms of their general background (all comparisons yielding *p* values above 0.2), including mean age (move group: *M* age = 24, s.d. = 3; control group: *M* age = 23*,* s.d. = 2), gender (43/53 female in the move group and 53/61 in the control group), years of education (move group: *M* = 16, s.d. = 1; control group: *M* = 17, s.d. = 1) and non-verbal reasoning as assessed through a progressive matrices test [28,29] (move group: *M* = 69%, s.d. = 20; control group: *M* = 65%, s.d. = 26). Parental education was higher in the move group (63% of participants who answered the question reported that one or both parents had completed higher education) relative to the control group (24% had parents with higher education; *p* < 0.001).

The two groups were also comparable in their language profile assessed at baseline through a questionnaire completed by both groups before the move group moved to the UK. All participants were recruited from a degree related to linguistics and/or education, typically a degree related to English. We focused on these degrees as we wanted our participants to already have at least an intermediate L2-English proficiency level. This allowed us to focus on changes in language environment and language use as opposed to large changes in proficiency in beginning language learners. All participants acquired L1-Mandarin from birth and started L2-English acquisition during childhood. They were more proficient in their L1 than L2, with all tests showing a significant difference between the languages. However, in most cases, participants had already reached at least an intermediate L2 proficiency level at baseline. The detailed language background measures at baseline are reported in table 1. This includes self-reports of language proficiency and objective proficiency measures including a short lexical decision task (LexTALE [30,31]) and a picture-naming task (based on [32]). Further proficiency tests (e.g. an interview and a vocabulary test) were included in the longitudinal sessions but not in the baseline. These are described in §2.3.

**Table 1.** Summary of objective and subjective measurements of Mandarin and English proficiency and use, measured in the baseline (pre-move) assessment for the move group and the control group. Groups did not differ significantly on any of the age of acquisition or proficiency measurements reported in this table (all *p* > 0.05). The only significant between-group difference was in terms of current language use as reported in the Language and Social Background Questionnaire (LSBQ; indicated with an asterisk, *p* < 0.05). The move group reported using English more often, potentially in preparation for their move to the UK. None of the other language use measures showed significant differences between groups. Across groups, L1 proficiency and use were significantly higher than L2 proficiency and use on all measures.

	move group (*n* = 53)			control group (*n* = 61)		
	mean	s.d.	min–max	mean	s.d.	min–max
**L2 age of acquisition^a^**						
*age start of acquisition*	8.2	2.4	3−14	8.4	2.0	3−13
*age of first L2 conversation*	10.6	3.5	5−20	11.2	3.0	5−20
*age of first L2 use outside classroom*	15.9	3.9	6−25	14.9	5.0	5−26
**LexTALE (0–100%)^b^**						
*L1-Mandarin*	94.1	9.5	57.5−100	96.8	4.4	85−100
*L2-English*	67.3	12.5	47.5−95	64.6	11.5	41.3−100
**picture naming test** **(0–100%)^c^**						
*L1-Mandarin*	96.8	3.6	83.1−100	94.6	9.7	50.8−100
*L2-English*	75.7	12.2	47.7−96.9	76.7	20.3	35.4−98.5
**L1-Mandarin self-rated proficiency (1–10)^d^**						
*speaking*	9.4	0.9	7−10	9.2	1.1	6−10
*understanding*	9.5	1.0	5−10	9.2	0.9	7−10
*writing*	8.5	1.5	4−10	8.0	1.6	4−10
*reading*	9.2	1.0	6−10	8.7	1.2	6−10
**L2-English self-rated proficiency (1–10)^d^**						
*speaking*	5.7	1.2	3−8	5.6	1.3	3−9
*understanding*	6.5	1.4	2−9	6.5	1.3	3−9
*writing*	5.6	1.1	3−8	5.8	1.4	3−9
*reading*	6.8	1.2	4−9	6.7	1.4	3−9
**LSBQ language use** **(1 = all English; 5 = all Mandarin)^d^**						
*childhood (home and school) use*	4.4	0.3	3.7−5	4.3	0.3	3.4−5
current use across contexts and people*	3.7	0.7	1.8−4.8	4.1	0.5	2.7−4.8
**language use** **(0–100% of the time)^d^**						
*L1-Mandarin*	81.5	15.3	40−100	84.3	10.8	50−99
*L2-English*	18.5	15.3	0−60	15.7	10.8	1−50
**language exposure** **(0–100% of the time)^d^**						
*L1-Mandarin*	82.3	17.4	30−100	80.2	18.6	0−100
*L2-English*	17.7	17.4	0−70	18.1	15.6	0−88
***time spent in a single-language environment^d^*** *(0–100% of the time)*	67.8	27.1	0−100	63.5	29.4	0−100

^a^ Fourteen participants in the move group and 26 participants in the control group indicated that they were not using English outside the classroom.

^b^ The L1-Mandarin LexTALE was developed after the start of the study and only completed by participants in the second wave (academic year 2022–2023; *N* move group = 27; *N* control group = 30). L2-English data are missing for three participants in the move group.

^c^ Due to technical difficulties or participants using the wrong language throughout the task, L1 data are missing for nine participants in the move group and five in the control group. L2 data are missing for six participants in the move group and 10 in the control group.

^d^ Data are missing for three participants in the move group. Data were complete in the control group, with the exception of the final measure (time spent in single-language environment), which could not be computed for two participants.

Table 1 also reports data regarding participants’ language use at baseline. In both groups, participants’ use of and exposure to L1-Mandarin was higher than to L2-English. Language use was assessed through the Language and Social Background Questionnaire (LSBQ) [33], which asks questions about language use in different contexts and with different people. We also asked participants to indicate their relative use of and exposure to each language on a 0–100% slider. Finally, we asked participants how much time they spent in single-language contexts as part of a questionnaire also asking about switching behaviours [13]. Further details about the participants’ switching behaviour are not reported here considering the focus on single-language contexts. However, this is described in more detail in a separate paper focusing on dual-language contexts and switching behaviours [26] (https://osf.io/a24xv/).

In addition to these baseline measures, we examined participants’ proficiency and use throughout the study. This is further reported and discussed in §3.3 (table 8), which confirms that participants in the move group indeed increased their L2 proficiency, L2 use and time spent in L2 single-language contexts in the UK compared to before they moved, while their time in L1 single-language contexts decreased. During the first testing wave, several COVID-19-related restrictions were still in place in the UK, but they mostly concerned quarantines after travelling, certain large-group size restrictions and recommended use of face masks. Participants in both testing waves followed classes in person and were able to meet and socialize with people in person. Furthermore, additional checks confirmed that participants’ reported daily-life L2 use and exposure while in the UK did not differ between the first testing wave (2021−2022, when some restrictions were still in place) and the second wave (2022−2023).

### 2.2. Design

Both tasks (verbal fluency and picture naming) included the same variables: the language in which participants had to respond (L1-Mandarin or L2-English), session (A or B), group (move group or control group) and language order. Language order referred to the language being used first (e.g. picture naming in Mandarin before picture naming in English) or second (e.g. Mandarin after English). In the verbal-fluency task, language order was manipulated between participants, with 47% of move group participants and 48% of control group participants starting the task in L1-Mandarin. With two exceptions, the language order was the same for a participant in the two sessions (i.e. participants who started in their L1 in session A also started the task in their L1 in session B). Manipulating language order between participants is common in the literature. However, in the picture-naming task, we manipulated it within participants across two blocks. This was easier to set up here as a proportion of pictures was repeated within participants. In contrast, for the verbal-fluency task, we did not want to repeat categories (to avoid order effects related to remembering exemplars fitting a semantic category), and we could not find enough comparable semantic categories to manipulate language order within-participant. Within the picture-naming task, in one block, participants completed the task in English first and Mandarin second. In the other block, the opposite language order was followed. The picture-naming task included both repeated and unrepeated items. Within the repeated items, we furthermore manipulated frequency of the picture names (high or low frequency) as an additional within-participant variable (see electronic supplementary material).

### 2.3. Materials

#### 2.3.1. Language control tasks

##### 2.3.1.1. Verbal fluency

In the verbal-fluency task, the following semantic categories were used: animals, body parts, clothes, fruits and vegetables, furniture and rooms, jobs, musical instruments, sports, vehicles and toys or buildings (in the first wave, we used toys, but this category was too difficult and therefore replaced with buildings in the second wave). It was not possible *a priori* to match the semantic categories for the number of possible Mandarin and English responses, but we did select only categories that were common and relevant in both cultures. Furthermore, we opted for semantic rather than phoneme fluency to avoid strong language-specific effects. Each participant completed the verbal-fluency task three times (before moving to the UK, in session A and in session B). In the baseline test before moving, they only completed one category per language. In sessions A and B (of main interest), they completed two categories in a row per language. Participants saw different categories per language and per session. The categories were counterbalanced (as to which session and which language they were used for) across participants. Unlike the picture-naming task, we deliberately did not repeat the categories across languages as participants could otherwise strongly benefit from having previously come up with semantic concepts in the same category. This furthermore allowed us to examine language control across the language more globally, as opposed to item/category-specific language control.

##### 2.3.1.2. Picture-naming task

**Figure 1.**
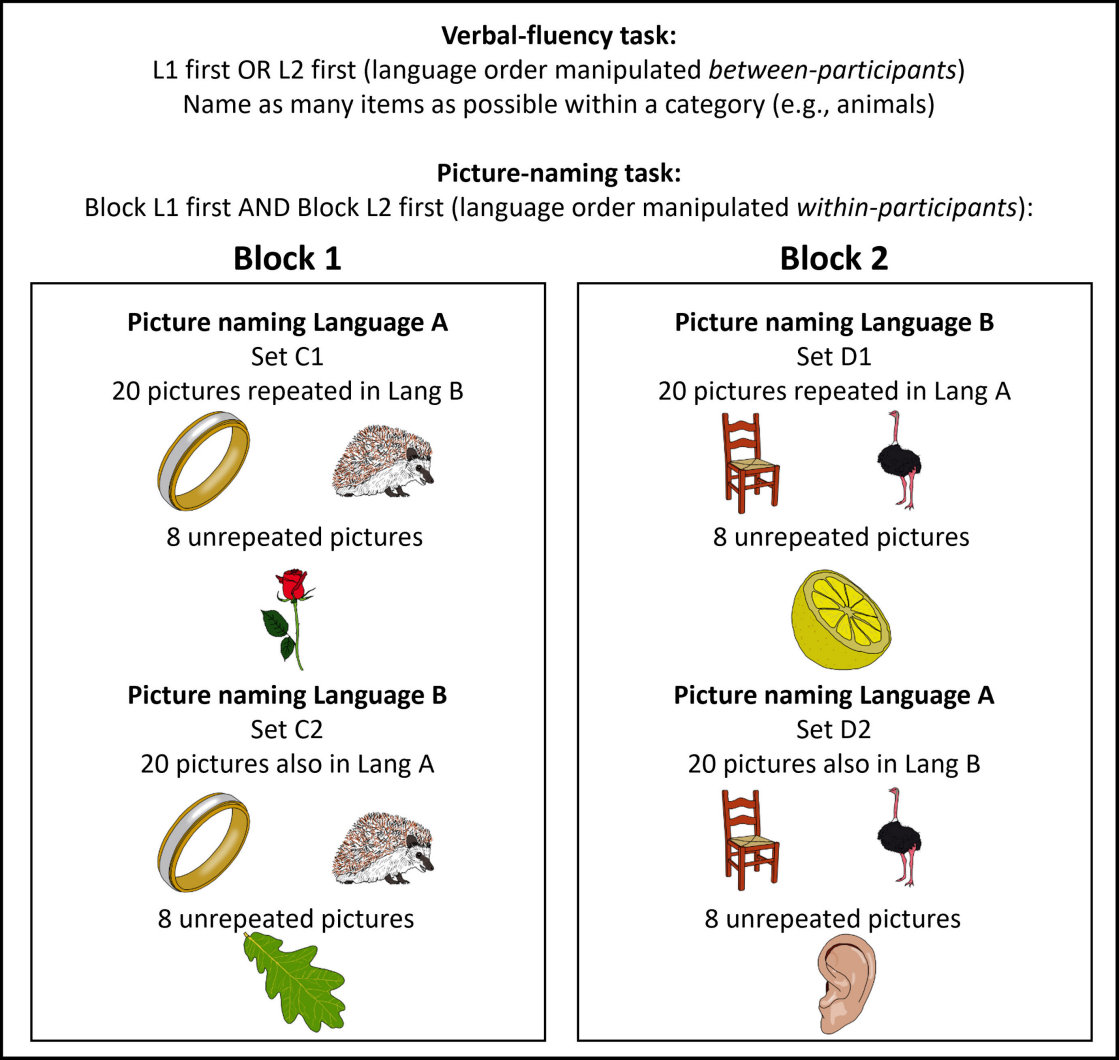
Overview of the two tasks, including a further description of the picture-naming task. The order of verbal-fluency or picture-naming tasks first was counterbalanced across participants. Within the picture-naming task, each participant completed both blocks, with one block starting in the L1 and the other block in the L2. Within each block, 20 pictures were presented once in each language part, and eight pictures were only presented in one of the languages. Different sets of stimuli were used across blocks and across sessions in the picture-naming task and across sessions and languages for the verbal-fluency task (counterbalanced across participants).

In the picture-naming task (see figure 1), one block was completed in the L1 first and L2 second and another block followed the opposite order. Participants named 28 pictures per language in each block (112 pictures in total per participant per session). Within each block, 20 pictures were repeated so that they were presented once in the L1 part and once in the L2 part (see OSF page for the full stimulus list and electronic supplementary material for frequency details). Per block, participants named an additional eight pictures in the L1 only and another eight unrepeated pictures in their L2 only. While the main analysis focused on the repeated pictures, these unrepeated items were included to examine lexicon-global language control effects too. Participants always saw different items in sessions A and B and different items in the L1-first versus L2-first blocks, with the combination of stimuli, blocks and sessions counterbalanced across participants.

### 2.3.2. Language background measures

#### 2.3.2.1. Age of acquisition

Age of L2-English acquisition was assessed through three questions: age when participants first started to learn English; age when they had their first conversation in English; and age when they first started to use English outside the classroom (where applicable to a participant).

#### 2.3.2.2. Language proficiency

Language proficiency was self-reported in each language in relation to speaking, understanding, writing and reading. We also included the following objective proficiency measures. The LexTALE test [30,31] is a short lexical decision task in which participants see letter or character strings and indicate if they form an existing word or not. LexTALE scores were computed with the formula provided by Lemhöfer & Broersma [30]: ((number of words correct/40 × 100) + (number of nonwords correct/20) × 100)/2. Receptive vocabulary was also assessed through a short vocabulary size test (L2-English only), based on [34] in which participants saw a word with an example sentence context, with four possible definitions. They had to choose the definition that best matched the word, and we computed their percentage of correct responses. Productive vocabulary was assessed through the BEST picture-naming test [32], in which participants named 65 non-cognate pictures per language. Again, percentage of accurately named words was computed. Finally, we assessed overall fluency through an interview [32] in which we asked the participants to give a short description of, for example, their favourite film or weekend plans. These were assessed by near-native speakers of English on a 1−5 scale. Low fluency was scored a 1 (participants know some words but cannot create a sentence) or 2 (participants can make simple sentences and express themselves a little but have many difficulties and make many mistakes). Intermediate fluency (participants are not completely fluent but can have a simple conversation with common mistakes and while showing difficulties with longer sentences) was scored as a 3 or 3.5, depending on the number of mistakes made. Advanced fluency was scored as a 4 (participants make some mistakes but are very fluent and can talk about almost every kind of topic) or 5 (native-like fluency). Finally, we also used a grammaticality judgement task, which was not further analysed for the purpose of the current study focusing on word production.

#### 2.3.2.3. Language use

Language use was measured through a modified version of the LSBQ [33], which has shown good test–retest reliability [35]. Participants were asked to indicate for a variety of contexts their language use on a scale from 1 (only English) to 5 (only Mandarin), with answer options 6 (all in another language) and 7 (context not applicable, e.g. if the participant did not have a partner) not used in the analysis. We computed mean scores for language use during childhood/teenage years and for current language use. General language use and exposure were also measured through two 0–100% slider scales asking participants to indicate their relative use of and exposure to English and Mandarin.

#### 2.3.2.4. Time spent in single-language contexts

Time spent in single- (and dual-)language contexts was assessed through the Bilingual Interactional Context Questionnaire (BICQ) [13]. Participants first reported how much time they spent at home, school, work and in other contexts. For each of those contexts, they then indicated the amount of time they spent in a single-language context (using only one language) and in two types of dual-language contexts (where two languages were used). The proportion of time spent in single-language contexts was computed relative to the time spent in each of the four overall contexts (e.g. home).

### 2.4. Procedure

Participants took part in three main sessions: baseline, session A and session B. The baseline session was completed online (using Gorilla.sc [36]), before moving to the UK. This questionnaire examined the participants’ background (as described in §2.1) and language profile (i.e. their age of acquisition, self-rated proficiency, LexTALE, BEST picture-naming, LSBQ, language use/exposure scores and BICQ, as well as other switching questionnaires not reported here). Sessions A and B included an in-person component and an online component. In the in-person part (which took about 1.5 h in total), participants completed a series of language control tasks. This included both single-language tasks, as described below, and dual-language switching tasks (reported in a separate paper, see https://osf.io/a24xv/). They also completed the interview, BEST picture-naming and grammaticality judgement tasks as proficiency measures. In the online component, they completed the other language-proficiency and daily-life language-use tasks and questionnaires as described above. Most participants in the move group completed the in-person part of session A within one or two weeks after moving to the UK. In the first testing wave, quarantine rules required participants to isolate for their first two weeks after arrival, which meant testing could not take place earlier. Participants in the move group completed the in-person session B on average 6.78 months after session A (s.d. = 7.82). A similar timeline was followed by the control group (session B on average happening 6.27 months (s.d. = 15.28) after session A). The time between sessions was significantly shorter in the control than in the move group (*p* < 0.001) due to COVID-related changes to the timings of the academic year. The online part of session A was completed on average 8.96 days after the in-person part. The online part of session B was completed on average 14.44 days after the in-person part.

Within each in-person session, the single-language tasks were always completed at the beginning of the study to avoid any influence of the dual-language tasks. Half of the participants completed the verbal-fluency task at the very beginning of the session while the other half started the session with the single-language picture-naming task. In the verbal-fluency task, participants were asked to provide as many exemplars as possible belonging to four different semantic categories. They completed two categories in a row per language (with language order counterbalanced). Following common procedures (e.g. [37]), participants were given 60 s per category.

The single-language picture-naming task had two blocks, to be able to manipulate language order within-participant. In one block, participants completed the task in their L1 first while they used the L2 first in the other block (with the first block starting in the L1 or L2 counterbalanced across participants). Within the picture-naming task, each picture stayed on the screen for 3 s, regardless of when a response was given. The next picture appeared after a fixation cross, which was shown for 500 ms. Participants named three practice trials (which differed from the experimental pictures) before each language block. The interview proficiency measure (ending with a final question that participants could answer in their language(s) of choice to return to baseline preferences) either separated the verbal-fluency and picture-naming tasks or was completed between the two blocks of the picture-naming task. This depended on the overall task order (i.e. whether participants started with verbal fluency or picture naming). For the other break between (blocks of) single-language production tasks, the research assistants had a brief conversation with the participants in their language(s) of choice.

We ensured the participants were tested in similar circumstances in the move and the control groups by having the two groups complete the exact same battery of tests and reading the same instructions on the screen in both languages. Additionally, trained Mandarin–English bilinguals led sessions A and B, and they received detailed instructions on how to run the study in both places.

### 2.5. Data analysis

To score the verbal-fluency task, we gave one point per word that belonged to the target semantic category. Words that could not clearly be identified as belonging to the category or not (e.g. ‘ice ball’ for the category ‘sports’), that could not be understood clearly from the recordings or transcriptions, as well as non-existing or inaccurate words (e.g. ‘cooker’ instead of ‘cook’ in the ‘jobs’ category) were excluded. Repeated words only received one point (i.e. no point for the repetition), but synonyms were counted separately (e.g. for the category ‘sports’, ‘soccer’ and ‘football’ would each receive a point). Note that some previous studies (e.g. [23]) have also examined the type of exemplars produced in the verbal-fluency task, in particular to analyse the presence of cognates. However, given that cognates are relatively less common in English and Mandarin, we did not examine the type of responses made. Furthermore, we also did not examine the frequency of the responses given, as frequency might change as a consequence of exposure to different items in different countries (e.g. more exposure to specific fruit types), rather than reflecting lexical processes.

In the picture-naming task, we scored as accurate (receiving a score of ‘1’) the target word, synonyms (e.g. ‘pants’ for the target word ‘trousers’) and words that were deemed acceptable descriptions of the target picture (e.g. ‘document’ was accepted for a picture representing a ‘letter’). Pictures that were named in the correct language but with a wrong word (e.g. ‘chair’ instead of ‘table’) were scored as a ‘2’. Pictures named in the wrong language (e.g. ‘table’ in Mandarin instead of English) received a score of ‘3’. If no response was given at all, it was scored as a ‘0’. Trials with a score of 0, 2 or 3 were counted as incorrect responses. RTs were scored in CheckVocal [38] using CheckFile).

Data were analysed in R (4.4.1; lme4 package v.1.1.35) using generalized linear mixed-effects analyses for the verbal fluency data (count scores, using the Poisson distribution) and accuracy in the picture-naming task (binomial distribution), and linear mixed-effects analyses for RTs in the picture-naming task. While we used two tasks to examine if (potential changes in) language control could be observed in both, we did not aim to compare them directly within one analysis due to the many differences between the two tasks.

The model for verbal fluency included the variables language (L2-English coded as −0.5; L1-Mandarin as 0.5); session (session A = −0.5; session B = 0.5); language order (language used first = −0.5; language used second = 0.5); and group (control = −0.5; move = 0.5). The dependent variable was the number of correct exemplars given. The final converging model included participant and item intercepts, participant slopes for language and session, and item slopes for language, language order, language × session, session × group, group × language order, language × session × group × language order. One participant from the control group was excluded from the analysis as they responded in the wrong language in both categories in session A. Two participants in the move group only completed one of the categories; for these two participants, only the completed category was included in the analysis.

For the picture-naming task, RTs were of main interest as previous studies have shown RT language order effects interacting with language, but not in terms of accuracy (e.g. [4]). However, given the high number of errors, we analysed accuracy too. Furthermore, given that participants were not familiarized with the pictures and because we included low-frequency pictures, we did not remove participants with lower accuracy (i.e. below 70%). The same variables were included as in the verbal fluency model. Our first, main, analysis focused on repeated items. The accuracy analysis converged with participant and item intercepts, all participant slopes and all item slopes apart from language order, language × language order, session × language order, group × language order, and the three-way interactions. The RT analysis converged with participant and item intercepts, all participant slopes and all item slopes apart from language × group, language × session, session × language order, group × language order, language × session × group, and session × group × language order. To further examine language order effects, we also analysed the (lower number of) unrepeated items.

For the picture-naming data, six participants were removed either because they completed the entire block in the wrong language (four participants) or because they did not respond at all/on only a few trials in one of the blocks, suggesting they did not focus on the task (two participants). Based on visual inspection, RTs were found not to be normally distributed, and we therefore used log RTs in the analysis. The RT analysis included correct responses only. The outlier removal procedure first removed trials below 300 ms and above 3000 ms (seven trials with timing issues) and then RTs falling 2.5 s.d. above/below the mean per participant and combination of conditions (0.6% of correct trials).

To foreshadow the results, neither the verbal-fluency task nor the picture-naming task showed a significant interaction between language, language order and session. Considering the smaller sample size than intended, we also conducted (not pre-registered) Bayesian analyses to quantify evidence for the null relative to the alternative hypothesis. These analyses were run using the BayesFactor package in R, using Bayesian ANOVAs on the mean scores per condition. We focused on the move group only to specifically examine evidence for or against any changes within this group. We compared the full model (all main effects and interactions) to a model including all main effects and two-way interactions but without the three-way interaction of interest. Bayes factors (BF_10_) above 1 would provide support for the alternative hypothesis of language control changing between sessions. Bayes factors below 1 would provide support for the null hypothesis of no changes in language control in the move group.

Finally, to address the final hypothesis regarding individual language profiles, we conducted a series of analyses using the move group data only. We ran three analyses for each task. The first analysis (analysis 1A) examined a potential relationship between language-profile predictors and language production in session B. The second analysis (analysis 1B) examined a potential relationship between language-profile predictors reported at session B and changes in language production between sessions A and B. The final analysis (analysis 2) examined a potential relationship between time-point changes in language-profile predictors and time-point (session) changes in language production. We first computed the models including all predictors. If there were any significant language-profile predictors, we then computed the model only including those significant predictors and compared it to the full model to determine the best-fitting model. If no significant difference was found in Akaike information criterion scores, the simpler model was preferred. Where relevant, we then also removed each significant language-predictor from the model. If removing the predictor did not make the model significantly worse, we again preferred the simpler model and removed that predictor from the final, best model. We report the results from the final best-fitting model (reporting values from analyses using maximum likelihood (ML) rather than restricted ML for the purpose of model comparison).

We included the following *z*-scored language-profile predictors: age of acquisition (composite score based on the three questions); L1- and L2-self-rated proficiency (composite score based on the four self-rated components); L2 objective proficiency (composite score based on the LexTALE (receptive), vocabulary size test (receptive), BEST picture-naming (expressive) and interview (expressive)); L1/L2 language use (composite score based on the current LSBQ questions and the general use/exposure questions, resulting in one relative score of L1 versus L2 use); and time spent in L1 versus L2 single-language contexts. The latter was constructed based on the LSBQ data, taking the percentage of contexts for which participants gave an ‘all English’ or ‘all Mandarin’ answer. We opted to work with these scores (as opposed to the BICQ ‘general time spent in single-language contexts’ score or a language entropy score reflecting time in single- versus dual-language contexts more generally) to differentiate between time spent in L1 versus L2 contexts specifically. L1 objective proficiency was close to ceiling for most participants and showed little variability between participants; these composite scores were therefore not included. The predictors were pre-registered and based on language-profile predictors of theoretical interest. The composite scores were computed as the mean of the standardized scores of the individual measures within each type of predictor. For both tasks, analyses 1A and 1B excluded five participants with one or more missing language-profile predictors in session B. Analysis 2 excluded seven participants with missing language-profile predictors in either/both the baseline assessment and session B.

## 3. Results

### 3.1. Verbal fluency

**Figure 2.**
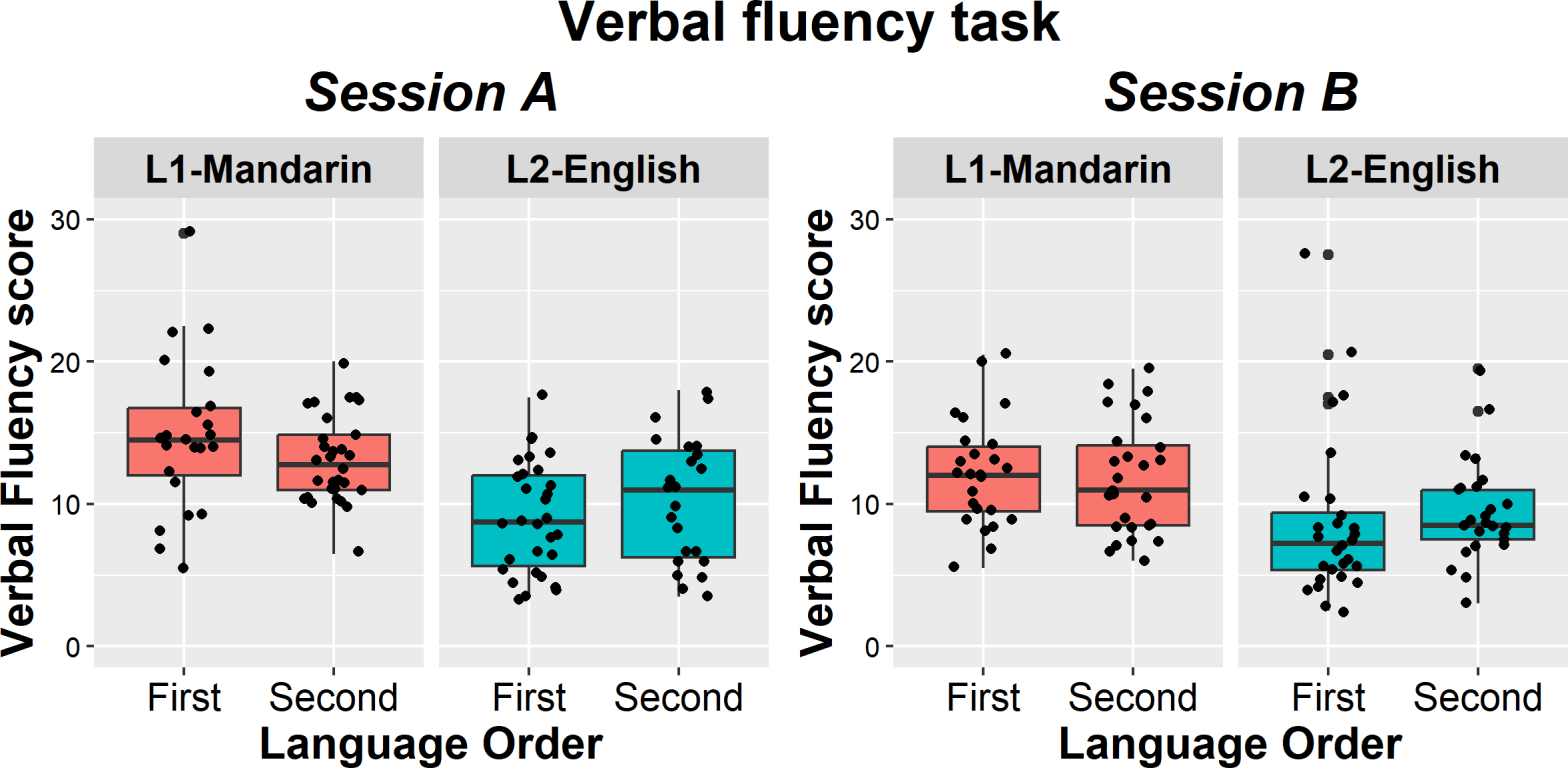
Verbal fluency results, move group only. The data for session A are shown on the left and for session B on the right. Within each plot, the left panel shows L1-Mandarin and the right panel L2-English. Within each language, the left boxplot shows the language being used first and the right boxplot the language being used second. Each individual participant is plotted as a black (jittered) dot. The horizontal line shows the median. Higher scores reflect better performance.

The full statistics for the verbal fluency analysis are reported in table 2, with table 3 showing the means per condition. Figure 2 shows the data from the move group. Across groups, there was a main effect of language, with participants producing more exemplars in their L1-Mandarin (*M* = 13.4 words, s.d. = 2.9) than in their L2-English (*M* = 8.7, s.d. = 3.1). Language also interacted with language order. L2-English benefited from being completed second, after the L1 (*M* = 9.2, s.d. = 3.0), compared to being completed first (*M* = 8.2, s.d. = 3.3; *β* = 0.112, SE = 0.056, *z* = 2.005, *p* = 0.045). The opposite pattern was observed in L1-Mandarin, which showed slightly lower performance when being completed second after the L2 (*M* = 13.0, s.d. = 2.7) compared to first (*M* = 13.8, s.d. = 3.1), although this did not reach significance (*β* = −0.069, SE = 0.036, *z* = −1.944, *p* = 0.052).

**Table 2.** Full results of the verbal fluency analysis. Significant fixed effects (*p* < 0.05) are indicated in bold.

predictor	estimate	SE	*z* value	***p* value**
**intercept**	**2.241**	**0.132**	**16.965**	**<0.001**
**language**	**0.486**	**0.070**	**6.910**	**<0.001**
session	−0.016	0.022	−0.706	0.480
group	0.034	0.038	0.897	0.370
language order	0.027	0.026	1.042	0.297
language × session	−0.040	0.049	−0.820	0.412
**language × language order**	**−0.195**	**0.075**	**−2.597**	**0.009**
**language × group**	**−0.151**	**0.049**	**−3.095**	**0.002**
**session × group**	**−0.121**	**0.051**	**−2.389**	**0.017**
session × language order	−0.013	0.043	−0.296	0.767
group × language order	−0.045	0.054	−0.834	0.404
language × language order × session	−0.031	0.087	−0.361	0.718
language × group × session	−0.123	0.084	−1.455	0.146
language × language order × group	−0.062	0.151	−0.411	0.681
group × language order × session	0.080	0.085	0.945	0.345
language × session × group × language order	0.141	0.195	0.724	0.469

**Table 3.** Scores (number of correct category exemplars produced) in the verbal-fluency task per language, language order (language ‘used first’ or ‘used second’), session and group.

	used first	used second
**move group**		
***language: L1-Mandarin***		
session A	14.8 (5.4)	13.1 (3.0)
session B	12.2 (3.8)	11.8 (3.9)
***language: L2-English***		
session A	9.0 (3.9)	10.3 (4.4)
session B	8.7 (5.7)	9.4 (3.5)
**control group**		
***language: L1-Mandarin***		
session A	13.8 (4.8)	13.6 (4.2)
session B	14.4 (5.0)	13.5 (4.2)
***language: L2-English***		
session A	7.7 (3.9)	8.6 (4.4)
session B	7.5 (3.9)	8.9 (4.6)

Of main interest for this study, we examined whether overall performance and language order effects changed between sessions. This was not the case. Language order effects (language × language order × session) did not differ significantly between sessions, suggesting proactive control did not change. In both sessions, only L2-English benefited from being used second, while L1-Mandarin performance was a little worse after L2 use (see table 3). Furthermore, across groups, overall performance did not differ significantly between sessions for both L1 (*M* session A = 13.8, s.d. = 4.4; *M* session B = 13.0, s.d. = 4.3) and L2 (*M* session A = 8.8, s.d. = 4.2; *M* session B = 8.6, s.d. = 4.5; no interaction language × session).

Some group differences were observed. Both groups showed significantly better L1 than L2 performance, but the difference between the L1 and L2 was larger in the control group (*M* L1 = 13.8, s.d. = 3.2; *M* L2 = 8.2, s.d. = 3.1) than in the move group (*M* L1 = 12.9, s.d. = 2.4; *M* L2 = 9.3, s.d. = 3.1; interaction language × group). However, this did not change between sessions (no interaction language × group × session), suggesting this was a baseline difference rather than a difference caused by the change in language environment. Overall performance also showed an interaction between session and group. This reflected that the move (but not control) group showed somewhat lower performance in session B than A overall (*β* = −0.062, SE = 0.031, *z* = −2.029, *p* = 0.042). Importantly, the group × session effect did not interact further with either language or language order, suggesting that any differences between groups did not concern language control specifically and did not affect the L1 or L2 differently.

Given that there was no significant interaction between language, language order and session (or group), the data suggest language control did not change in the move group between sessions. To be sure, we also ran Bayesian analyses with the mean verbal fluency performance of the move group only. The data were found to be around three times more likely to be observed under the null model (no change in language control) than under the full model (including a change in language control, BF_10_ = 0.331, error = 1.68%).

As a final check, considering some group differences were observed in both sessions, we also examined the short verbal-fluency task that participants completed in the baseline questionnaire (*n* = 57 in the control group and 48 in the move group). Both groups showed higher L1-Mandarin than L2-English performance in the baseline assessment (move group: *M* L1 = 15.8, s.d. = 6.0; *M* L2 = 12.9, s.d. = 6.3; control group: *M* L1 = 17.4, s.d. = 6.0; *M* L2 = 11.8, s.d. = 6.9; *β* = 0.290, SE = 0.092, *z* = 3.141, *p* = 0.002). The pattern of the move group showing a smaller L1–L2 difference than the control group was present at baseline too, but did not reach significance (*β* = −0.141, SE = 0.094, *z* = −1.498, *p* = 0.134). The two groups did not differ significantly at baseline in terms of overall score (*β* = 0.026, SE = 0.074, *z* = 0.348, *p* = 0.728) either. Although the baseline assessment was not set up to study language order effects, it should be pointed out that, for both languages, participants showed a higher performance when the language was completed second, with no difference between the languages (*β* = −0.036, SE = 0.167, *z* = −0.217, *p* = 0.828). This is potentially related to only one category being used and participants still getting used to the task (and thus benefiting from task repetition/practice in both languages).

### 3.2. Single language picture naming

#### 3.2.1. Accuracy

The full statistics from the picture-naming accuracy analysis can be found in table 4. The accuracy means per condition are provided in table 5. The main analysis (with repeated items) firstly showed a main effect of language, reflecting higher performance in L1-Mandarin (*M* = 91.8%, s.d. = 6.7) than in L2-English (*M* = 62.5%, s.d. = 12.4). There was also a main effect of language order. Performance was better when a language was used second, in which case participants had already seen the same pictures (*M* used second = 78.4%, s.d. = 8.1; *M* used first = 75.9%, s.d. = 9.4). Of interest for the current study, this did not interact with language, reflecting a similar increase in L1-Mandarin (*M* increase = 2.3%, s.d. = 9.3) and in L2-English (*M* increase = 2.6%, s.d. = 10.5) when the language was used second.

**Table 4.** Full results of the picture-naming accuracy analysis (repeated items only). Significant fixed effects (*p* < 0.05) are indicated in bold.

predictor	estimate	SE	*z* value	***p* value**
**intercept**	**2.447**	**0.238**	**10.286**	**<0.001**
**language**	**2.675**	**0.261**	**10.239**	**<0.001**
session	−0.181	0.096	−1.873	0.061
**group**	**0.494**	**0.200**	**2.469**	**0.014**
**language order**	**0.332**	**0.066**	**5.053**	**<0.001**
**language × session**	**−0.640**	**0.143**	**−4.481**	**<0.001**
language × language order	0.135	0.144	0.941	0.347
**language × group**	**−1.008**	**0.241**	**−4.189**	**<0.001**
session × group	0.088	0.183	0.481	0.631
session × language order	−0.138	0.134	−1.035	0.300
**group × language order**	**0.444**	**0.131**	**3.393**	**<0.001**
language × language order × session	0.110	0.330	0.334	0.738
language × group × session	−0.061	0.272	−0.223	0.824
**language × language order × group**	**0.894**	**0.287**	**3.117**	**0.002**
group × language order × session	−0.082	0.271	−0.303	0.762
language × session × group × language order	−0.326	0.718	−0.454	0.650

**Table 5.** Table showing percentage correct in the picture-naming task by group, language, session and language order. These percentages are collapsed across high-frequency repeated, low-frequency repeated and unrepeated pictures.

	used first	used second
**move group**		
***language: L1-Mandarin***		
session A	90.6% (14.0)	94.7% (5.4)
session B	88.4% (18.4)	92.7% (8.2)
***language: L2-English***		
session A	65.6% (14.8)	68.1% (13.0)
session B	69.3% (13.7)	70.5% (16.8)
**control group**		
***language: L1-Mandarin***		
session A	92.0% (6.3)	92.9% (6.9)
session B	89.9% (12.4)	90.3% (8.2)
***language: L2-English***		
session A	54.2% (14.3)	56.4% (13.3)
session B	56.1% (16.4)	57.9% (17.9)

No interaction was observed with session, language and language order (or group), suggesting language order effects did not change after a change in language environment either (see table 5). There was an interaction between language and session. Performance in L2-English did not change significantly between sessions (*M* increase = 2.0%, s.d. = 14.1; *β* = 0.139, SE = 0.099, *z* = 1.410, *p* = 0.159), while there was a decrease in L1-Mandarin performance between sessions (*M* decrease = −2.9%, s.d. = 10.9; *β* = −0.508, SE = 0.145, *z* = −3.499, *p* < 0.001). However, session effects did not interact with group, suggesting any language changes across sessions were not specifically related to a change in language environment.

Finally, there was a main effect of group, with overall performance in the control group (*M* = 74.7%, s.d. = 8.1) being lower than performance in the move group (*M* = 79.7%, s.d. = 7.5). This interacted with language, as performance was comparable in L1-Mandarin but lower in L2-English for the control than move group (see table 5). Group also interacted with language and language order. The move group showed a larger benefit of picture repetition (language being used second) than the control group (see table 5). In the move group, this concerned the L1-Mandarin in particular, which benefited more from being used second than the L2-English (*β* = 0.624, SE = 0.166, *z* = 3.771, *p* < 0.001). In contrast, in the control group, L2-English benefited somewhat more from pictures being repeated than L1-Mandarin, although not significantly (*β* = −0.312, SE = 0.160, *z* = −1.952, *p* = 0.051). Importantly, however, this did not interact with session in either group, suggesting any language differences in terms of language order did not change between sessions.

Finally, we also included a small set of unrepeated items, to examine any language order effects in the absence of picture repetition. Similar to the repeated items, performance was significantly better in L1-Mandarin than in L2-English (*β* = 2.495, SE = 0.233, *z* = 10.700, *p* < 0.001). The main effect of language order was significant (*β* = 0.192, SE = 0.080, *z* = 2.409, *p* = 0.016). Even though these specific pictures were not repeated, overall performance was better when the language was used second. Although this appeared somewhat more strongly the case for L1-Mandarin (*M* increase = 2.5%, s.d. = 12.9) than for L2-English (*M* increase = 0.2%, s.d. = 14.8), the interaction between language and language order did not reach significance (*β* = 0.368, SE = 0.191, *z* = 1.929, *p* = 0.054).

There was no interaction between language, language order and session (*β* = −0.040, SE = 0.389, *z* = −0.104, *p* = 0.917). In line with the previous analysis, language interacted with session (*β* = −0.355, SE = 0.167, *z* = −2.125, *p* = 0.034), reflecting an increase in L2-English performance (*p* = 0.012) but no change in L1-Mandarin performance between sessions (*p* = 0.720).

Overall performance was also significantly better in the move than in the control group (*β* = 0.757, SE = 0.190, *z* = 3.986, *p* < 0.001), in particular in L2-English (interaction language × group: *β* = −0.986, SE = 0.257, *z* = −3.833, *p* < 0.001). All other main effects and interactions were not significant (all *p* > 0.19), including no interactions between group and session.

#### 3.2.2. RT analyses

**Figure 3.**
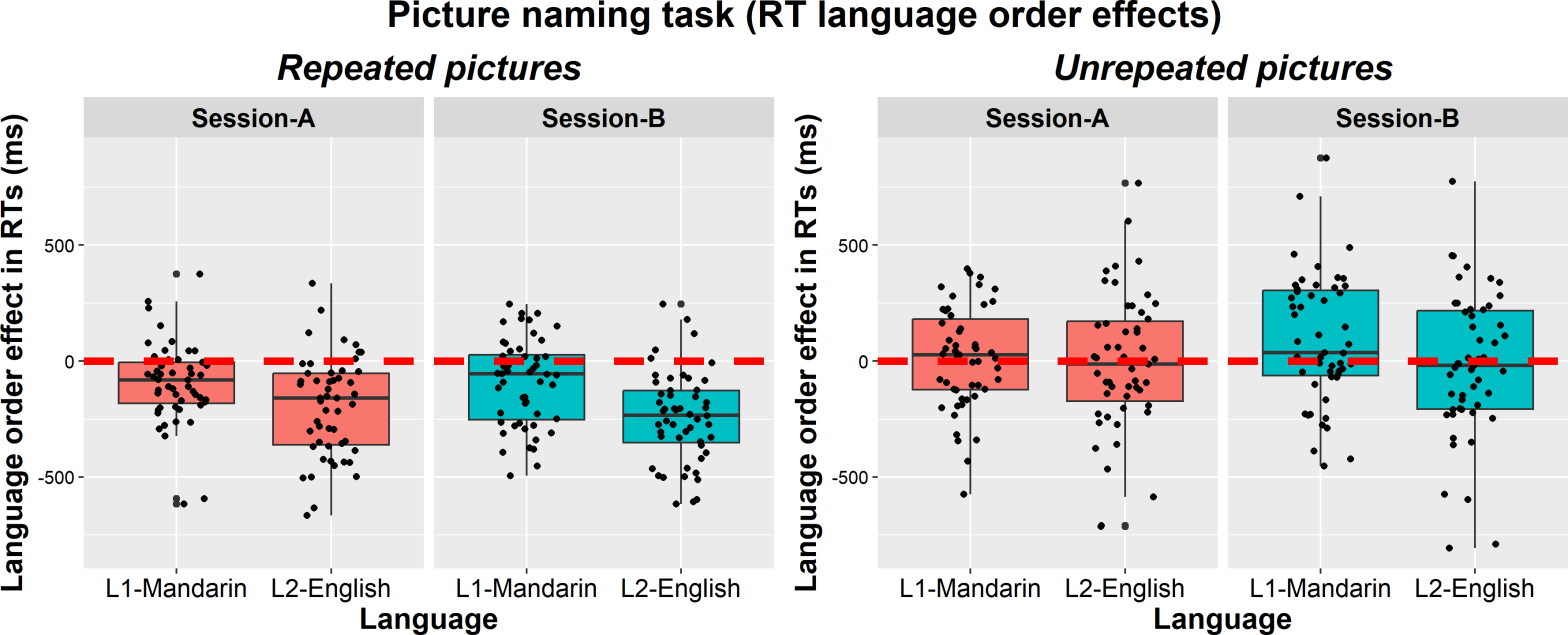
Results from the picture-naming task, showing the RT language order effects. For each participant, the language order effect was computed per language and session as the RT difference between that language being used second and that language being used first. The left plot shows the repeated pictures, and the right plot the unrepeated pictures. Within each plot, the left panel shows session A and the right panel session B. Within each panel, the left boxplot refers to the L1 and the right boxplot to the L2. Each individual participant is plotted as a black (jittered) dot. The red dashed line shows the zero line. Medians (black horizontal line) above the red line show poorer (slower) performance when the language is used second. Medians below the red line show facilitation when used second, with more negative scores showing larger facilitation.

Table 6 presents the full statistics for the RT analysis while the RT means by condition are shown in table 7. Figure 3 shows the results from the move group, computed as the language order effect per language (rather than the mean RTs by condition). A few participants did not have trials left in all combinations of conditions after error and RT outlier removal. Where relevant when computing means reported below, these participants were not included. The analyses reported below did include these participants but further analyses excluding them showed similar findings.

**Table 6.** Full results of the picture-naming RT analysis. Significant fixed effects (*p* < 0.05) are indicated in bold.

predictor	estimate	SE	*t* value	***p* value**
**intercept**	**7.141**	**0.019**	**383.958**	**<0.001**
**language**	**−0.105**	**0.017**	**−6.169**	**<0.001**
session	0.003	0.008	0.398	0.692
**group**	**−0.044**	**0.020**	**−2.143**	**0.034**
**language order**	**−0.113**	**0.010**	**−10.978**	**<0.001**
language × session	0.006	0.013	0.445	0.657
**language × language order**	**0.094**	**0.017**	**5.541**	**<0.001**
**language × group**	**0.088**	**0.019**	**4.623**	**<0.001**
session × group	0.027	0.017	1.624	0.108
session × language order	−0.012	0.012	−1.024	0.308
group × language order	−0.025	0.013	−1.904	0.060
language × language order × session	0.023	0.024	0.947	0.347
language × group × session	0.025	0.025	0.976	0.331
language × language order × group	0.031	0.027	1.147	0.254
group × language order × session	−0.011	0.023	−0.489	0.626
language × session × group × language order	0.010	0.047	0.203	0.839

**Table 7.** Table showing RTs (across repeated and unrepeated items) in the picture-naming task by group, language, session and language order (language ‘used first’ or ‘used second’).

	used first	used second
**move group**		
***language: L1-Mandarin***		
session A	1295 (185)	1231 (183)
session B	1317 (188)	1281 (202)
***language: L2-English***		
session A	1396 (186)	1257 (174)
session B	1433 (185)	1265 (185)
**control group**		
***language: L1-Mandarin***		
session A	1311 (156)	1245 (186)
session B	1270 (161)	1235 (182)
***language: L2-English***		
session A	1472 (186)	1353 (170)
session B	1491 (186)	1353 (175)

There was a main effect of language, with faster responses in L1-Mandarin (*M* = 1244 ms, s.d. = 138) than in L2-English (*M* = 1313 ms, s.d. = 149). Of main interest for this study, there was a language order effect that interacted with language. Both languages showed faster responses when they were used second (*p* < 0.001), but this benefit was larger for L2-English (*M* benefit = −188 ms, s.d. = 159) than L1-Mandarin (*M* benefit = −85 ms, s.d. = 128).

The language order effects and the difference between the languages were comparable for session A (language order effect: *M* L1 = −96 ms, s.d. = 179; *M* L2 = −159 ms, s.d. = 224) and session B (*M* L1 = −80 ms, s.d. = 179; *M* L2 = −214 ms, s.d. = 199; no interaction between language × language order × session). Overall RTs, and language differences, also remained stable across sessions (no interactions with session; see table 6).

Finally, there were some group differences but they did not relate to the language order effect of interest. In the L2, the move group responded faster than the control group (*M* move = 1277 ms, s.d. = 155; *M* control = 1346 ms, s.d. = 137; *β* = −0.090, SE = 0.023, *t* = −3.859, *p* < 0.001). In contrast, in the L1, there was no significant difference (*β* = 0.001, SE = 0.021, *t* = 0.024, *p* = 0.981) between the control group (*M* = 1239 ms, s.d. = 129) and the move group (*M* = 1250 ms, s.d. = 147).

We also ran an analysis examining the unrepeated items, to further examine the facilitation observed for the repeated items in relation to language order (see figure 3). The unrepeated items also showed a language effect (*β* = −0.181, SE = 0.019, *t* = −9.598, *p* < 0.001) that interacted with language order (*β* = 0.040, SE = 0.015, *t* = 2.616, *p* = 0.012). In L2-English, there was no significant language order effect (*β* = −0.008, SE = 0.012, *t* = −0.661, *p* = 0.510), and if anything, this was going in the direction of facilitation (*M* = −15 ms, s.d. = 250). In contrast, in L1-Mandarin, RTs were significantly slower when the task was completed second in that language (*M* = 40 ms, s.d. = 171; *β* = 0.030, SE = 0.009, *t* = 3.358, *p* = 0.001). These language order effects did not interact with group or session (all *p* > 0.1).

There was also a main effect of group (*β* = −0.054, SE = 0.019, *t* = −2.750, *p* = 0.007) and an interaction between group and language (*β* = 0.120, SE = 0.023, *t* = 5.124, *p* < 0.001). This further interacted with session (*β* = 0.075, SE = 0.031, *t* = 2.414, *p* = 0.020). This interaction reflected different patterns in terms of language differences across sessions in the two groups. In the move group, the RT difference between the L1 and L2 became smaller between sessions (*M* difference session A = 147 ms, s.d. = 167; *M* difference session B = 102 ms, s.d. = 200), although this did not reach significance (*β* = 0.037, SE = 0.021, *t* = 1.773, *p* = 0.082). This was mostly related to L1 responses showing a larger increase in RTs in the second session than the L2 responses. In contrast, although again not significant (*β* = −0.040, SE = 0.023, *t* = −1.776, *p* = 0.081), in the control group, the RT difference between the L1 and L2 became larger between sessions (*M* difference session A = 237 ms, s.d. = 246; session B = 309 ms, s.d. = 236). This was the result of L1 responses being slightly faster in session B and L2 responses being slightly slower in session B in this group (see table 7 for this pattern across repeated and unrepeated items).

Similar to the verbal fluency analysis, given that there was no significant interaction between language, language order and session (or group), we ran a Bayesian analysis to further examine if language control indeed did not change in the move group between sessions. We only did this for the RT analyses as accuracy did not show a language × language order interaction to start with. The RT data for repeated pictures were found to be around 4.5 times more likely to be observed under the null model (no change in language control) than under the full model (including a change in language control, BF_10_ = 0.224, error = 1.63%). The RT data for the unrepeated pictures were around 3 times more likely to be observed under the model without a change in language control (BF_10_ = 0.338, error = 1.57%).

### 3.3. Individual differences

The final analyses examined a potential relationship between (changes in) the production tasks and individual differences in language profile in the move group. Table 8 presents the language-profile predictor means at baseline and in session B. Within the move group, participants’ self-reported L1 proficiency decreased between sessions while their self-reported and objective L2 proficiency increased. Participants spent significantly more time in L2 single-language contexts (and dual-language contexts) after their move, while time in L1 single-language contexts decreased. Although we focus on reporting the move group results here (as they were of main interest and expected to change), checks of the control group’s language profile confirmed that their L2 proficiency and L2 use indeed did not increase throughout the study.

**Table 8.** Overview of the language profile composite scores (per predictor included in the individual-difference analysis) at baseline and in session B. All scores apart from age of acquisition are reported on a scale of 0–100%. For ‘language use’, a higher score reflects more L1-Mandarin use, while a lower score reflects more L2-English use. For the L2 objective proficiency composite score, two included measures (interview and vocabulary size) were collected at session A rather than in the pre-move baseline assessments. For the purpose of this comparison, this table only includes the 46 participants in the move group who completed all relevant language-profile questionnaires at baseline and in session B.

language profile predictor	baseline	session B	comparison
age of acquisition (years)	11 (3)	N/A	N/A
L1 self-reported proficiency	92% (9)	87% (12)	*t*(45) = 2.696, *p* = 0.010
L2 self-reported proficiency	62% (9)	66% (10)	*t*(45) = −3.055, *p* = 0.004
L2 objective proficiency	67% (8)	70% (7)	*t*(45) = −3.089, *p* = 0.003
language use	75% (13)	55% (12)	*t*(45) = 7.741, *p* < 0.001
time in L1 single-language contexts^a^	33% (22)	14% (11)	*t*(45) = 5.574, *p* < 0.001
time in L2 single-language contexts	8% (13)	13% (12)	*t*(45) = −2.309, *p* = 0.026

^a^ Time in L1 and L2 single-language contexts does not add up to 100% as these scores reflect time in contexts that only use the L1 or L2. The remaining time was spent in dual-language (L1 and L2) contexts where both languages are used. A decrease in L1 single-language contexts therefore also reflects an increase in dual-language contexts.

#### 3.3.1. Verbal-fluency task

Although we pre-registered that the individual-difference analyses would include language order effects for both tasks, given that language order was manipulated between participants in the verbal-fluency task, these analyses could not assess language order effects within participant. They were therefore run to only assess a potential relationship with language, not including language order.

The first analysis (1A) examined a potential relationship between verbal-fluency scores and language profile in session B. The best model included only one language-profile predictor: self-rated L1 proficiency (*β* = −0.081, SE = 0.030, *z* = −2.668, *p* = 0.008). Participants who reported a higher L1 proficiency produced fewer exemplars in the verbal-fluency task in general. This did not interact with language. None of the other predictors were significant in the full model (all *p* > 0.15). The second analysis (1B) examined whether the language profile reported in session B related to potential individual changes in the verbal-fluency task between sessions. No significant language experience predictors were found (all *p* > 0.075). Similarly, analysis 2 (examining whether changes in daily-life language experiences related to individual changes in the verbal-fluency task) showed no significant language-profile predictors (all *p* > 0.24).

#### 3.3.2. Picture-naming task

In the picture-naming task, language order was manipulated within participants and could therefore be examined in relation to language profile too. The first individual language profile analysis (1A) examined a potential relationship in the final session. The best model included two language-profile predictors. First, there was a significant relationship with L2 objective proficiency (*β* = −0.034, SE = 0.015, *t* = −2.200, *p* = 0.033), reflecting faster overall responses (across languages) in people with a higher L2 proficiency. Second, there was an interaction between language and time spent in L1 single-language contexts (*β* = −0.035, SE = 0.015, *t* = −2.299, *p* = 0.026). Participants who spent more time in L1 single-language contexts showed a larger RT difference between the L1 and L2, reflecting relatively slower L2 naming and faster L1 naming. None of the language-profile predictors interacted significantly with language order or with language order × language.

Analysis 1B examined whether any individual changes in production tasks between sessions related to individuals’ language profiles in session B. The best model included one language-profile predictor. Frequency of language use interacted with language and session (*β* = −0.044, SE = 0.017, *t* = −2.579, *p* = 0.013). This reflected that in L1-Mandarin (*p* = 0.024), but not in L2-English (*p* = 0.427), language use frequency interacted with RT changes across sessions. Participants who used L1-Mandarin less in their daily life were more likely to show an increase in L1 RTs between sessions.

Analysis 2 examined potential relationships between changes in single-language production RTs and changes in language profile. None of the language-profile predictors were significant (all *p* > 0.1).

Following Casado *et al*. [11], we conducted one final exploratory analysis. Our pre-registered analyses focused on daily-life language experiences. However, Casado *et al*. found a relationship between language order effects and a bilingual’s lexical retrieval speed in each language within the task. We therefore computed (across sessions) the difference between L1 and L2 RTs (L1 RTs − L2 RTs) when that language was used first, and examined the correlation with the participants’ L1 order effect. Across all items, there was a significant correlation (*r*(106) = 0.281, *p* = 0.003). This reflected that participants with a larger (negative) L1–L2 difference (i.e. more unbalanced in speed of lexical retrieval) showed a more negative/less positive L1 order effect. However, this correlation was not significant when only considering the unrepeated items, where facilitation due to item repetition could not explain the results (*r*(106) = 0.111, *p* = 0.252).

## 4. General discussion

This study examined the way bilinguals apply proactive language control during language production in different single-language tasks (verbal fluency and picture naming), focusing on potential changes in language control after a change in language environment. We manipulated language order (the task being completed in that language first, or after completing the task in the other language) as a measure of language control. Both tasks showed that the L1 and L2 were impacted differently by language order, which suggested that the bilinguals were proactively applying language control in both tasks and in particular while using the L2. We furthermore examined whether this language control adapted to the language environment bilinguals lived in, through a longitudinal analysis of Mandarin–English bilinguals moving from China to the UK. Neither task showed changes in terms of language-control measures between the testing sessions, suggesting language control did not change after moving to a new L2 environment.

### 4.1. Proactive language control

Both tasks showed different language order effects for the L1 and the L2. L2 performance was either better after doing the task in the L1 first (verbal fluency and repeated picture RTs) or not significantly influenced by language order (unrepeated picture RTs). L1 performance benefited less from doing the task after the L2 compared to being used first (repeated picture RTs) or was worse if the L2 was used first (unrepeated picture RTs and verbal fluency, although the latter did not reach significance). These findings suggest bilinguals applied more language control while doing the task in their L2. This appeared to be the case both when pictures were repeated (item-specific control) as well as when pictures or categories were not repeated (lexicon-global control). Importantly, we also observed language differences in order effects when language order was manipulated *within* participants, while previous studies have typically manipulated language order between participants (e.g. [4], but see [7]).

These patterns are consistent with language order effects observed across the literature (e.g. [4,7,10]). Similar to those studies, and in particular when the same items are repeated across tasks, the L2 was found to benefit from being used after the L1. This likely reflects facilitation effects related to easier lexical retrieval after previous exposure as well as task practice. When the exact same pictures were used and considering participants were not familiarized with these pictures before the study, the L2 in particular appeared to benefit from previous exposure to those pictures. This was especially the case for lower-frequency L2 items (see electronic supplementary material). This suggests L2 words that are more difficult to retrieve benefit especially from previous exposure. However, even when items were not repeated, as was the case in the verbal-fluency task, the L2 appeared to benefit from task practice. In contrast, the L1 either benefited less from item repetition or, when items/categories were not repeated, showed poorer performance when used after the L2. For repeated picture naming, the L1 still benefited from previous exposure to these pictures, but less so than the L2. This finding on its own could suggest the L1 simply benefits less from practice or repetition. However, the *unrepeated* items in the picture-naming task, as well as the verbal-fluency data, did not show less facilitation for the L1 but rather a negative effect: L1 performance was *worse* when used after the L2.

This negative effect when the L2 preceded the L1 is commonly interpreted to reflect language control, which is applied most strongly during L2 use. This control could be in the form of inhibition, with the (dominant) L1 requiring more inhibition during L2 production than vice versa. As a consequence, when the L1 has to be used after the L2, more time might be needed for L1 words to be reactivated (e.g. [4,6]). This could result in poorer performance when there are no/smaller benefits of task repetition or, when items are repeated, can reduce repetition facilitation. However, other forms of language control can explain these data too. Branzi *et al*. [7] propose an alternative explanation, which argues that participants use language control in the form of L2 over-activation. According to this interpretation, poorer L1 performance when being used second could be explained through bilinguals over-activating the L2 words during L2 production. Such L2 over-activation could carry over and result in more interference with the L1 when the L1 has to be produced next. In both cases, bilinguals are argued to (potentially proactively) apply language control, through either L1 inhibition or L2 over-activation to manage competition between languages, in particular to manage the stronger competition coming from the L1.

These language order effects were observed in both verbal-fluency and picture-naming tasks, suggesting they arise regardless of the task demands on lexical selection. Indeed, these language order effects have been found in the literature in both picture-naming [4,7] and verbal-fluency tasks [10]. However, one previous study compared both within the same participants [10] and only found order effects in the verbal-fluency task. In line with the literature (e.g. [4]), our data furthermore only showed these effects for picture-naming RTs but not for accuracy, suggesting that language control specifically affects the *speed* with which words can be retrieved. Accuracy scores were more likely to reflect participants not knowing the word (especially in the L2) rather than an impact of lexical retrieval and control. Both languages therefore showed facilitation effects in terms of accuracy.

Finally, our study provides evidence that this language control is also applied over the lexicon more globally (as observed when pictures are not repeated and when different semantic categories are used) and not purely locally, over specific translation equivalents (see also [7]). This suggests that this control can be applied proactively, in anticipation of potential language competition across the lexicon. Previous research [10] observed that only certain types of bilinguals might apply language control globally (across the lexicon). In their study, only bilinguals living in an L2-dominant environment (i.e. bilinguals who potentially need to use more L1 control to use the L2) showed lexicon-global language order effects differing between the L1 and L2, while bilinguals in an L1-dominant environment did not. However, those two groups also differed in various other ways, including the languages spoken (Dutch–English versus Mandarin–English). In our study, within bilinguals speaking the same languages, language order effects (interaction language order × language) were comparable for bilinguals living in an L1-dominant environment (control group) and those living in an L2-dominant environment (move group). This suggests global language control might be applied similarly by bilinguals speaking the same languages but living in different types of language environments. Furthermore, our analysis looking at a relationship with language profile showed no significant influence of language proficiency and language use on (L1–L2 differences in) language order effects. Both language proficiency and language use (time in L1 single-language contexts) were related to overall performance, with participants with higher self-rated L1 proficiency producing fewer verbal-fluency exemplars, participants with a higher L2 proficiency being faster overall in the picture-naming task and participants spending more time in L1 single-language contexts showing a larger L1–L2 RT difference, related to relatively slower L2 and faster L1 naming. However, these relationships corresponded to lexical access more generally and did not interact with language order effects, suggesting that language control was not modulated by the language profile predictors measured. As a final check, we also ran analyses similar to Casado *et al*. [11]. These analyses considered participants’ speed of lexical retrieval within the task itself, as opposed to their language experiences. In line with Casado *et al*., we found a relationship between participants’ L1–L2 baseline differences and the L1 order effect. More unbalanced bilinguals (larger L1–L2 RT difference) showed a less positive L1 language order effect in the picture-naming task (i.e. less facilitation). This can reflect that these participants applied more language control over the L1 while using the L2. However, no significant relationship was observed for the *unrepeated* picture L1 order effect, suggesting the observed correlation when including repeated pictures could reflect an influence of picture repetition rather than a relationship with language control.

### 4.2. Changes in language environment

In terms of effects of moving to a new language environment, none of the analyses showed significant interactions between language, language order and session (or group). This was supported by the Bayesian analyses. This suggests there were no between-session changes in terms of language control (i.e. language order differences between languages). Furthermore, while some small differences were found between groups in some of the analyses, those remained stable across sessions and did not relate to language control. Together these findings suggest that (i) language control did not differ between the two groups living in different language environments and (ii) language control did not change after moving to a new language environment. Furthermore, no relationships between language control and individual differences in (changes in) language experiences were observed either.

These findings suggest that language control might not adapt to the overall language environment a bilingual lives in. Specifically, the proactive control bilinguals apply during single-language contexts does not seem to change when bilinguals move from an L1-dominant environment (requiring less L2 use and therefore hypothesized to involve less control to avoid L1 interference) to an environment that requires more L2 use and was therefore predicted to introduce different levels of L1 interference and control. Rather than changing in response to the overall environment, the type of language control needed (e.g. proactive control in a single-language context) might adapt rapidly to the specific immediate language context a bilingual is communicating in. Bilinguals can frequently change their immediate language context on a daily basis, for example, by going from making a phone call to their parents back home to speaking with an English classmate. Language control has been shown to flexibly adapt to these immediate language contexts (e.g. [26,39]). It is possible that these constant adaptations to the varying immediate contexts result in bilinguals not having one ‘overall’ language environment that their control can adapt to. Indeed, within the adaptive control hypothesis [5], three core immediate language contexts are defined that vary in their language control demands. The data from the current study, together with [26] examining different types of switching contexts, support the adaptive control hypothesis in terms of language control differing depending on the immediate context a bilingual is in. The data question, however, whether such flexibility extends beyond the immediate context to also showing adaptation to the more general, overall language environment. This also has consequences for the large literature examining potential relationships between bilingualism, bilinguals’ daily-life language environment and their executive control (following the adaptive control hypothesis [5]). These studies are based on the underlying hypothesis that language environment influences language control, and therefore potentially also performance on executive control tasks. However, if overall language environment does not (strongly or causally) influence language control, large impacts on executive control may not be expected either.

Previous studies that did suggest that performance of bilinguals in single-language contexts can differ depending on the language environment they live in typically compared different groups that differ in other aspects such as language proficiency (e.g. [17,18]). Furthermore, these studies did not manipulate language control, even if the results are explained through language control. For instance, although Linck *et al*. [18] concluded that worse L1 performance in participants living in an L2 environment was likely related to L1 inhibition, their verbal-fluency task assessed overall performance related to lexical access and did not include measures tapping into language control. Indeed, Baus *et al*. [23] concluded changes observed in their study were more likely related to changes in language use than to changes in control.

These studies [18,23] did show differences or changes in terms of lexical access, although they were not observed consistently across all tasks, measures or types of words [23]. In our study, changes in terms of overall performance were rare and not associated with a change in language environment specifically. The verbal fluency task showed poorer performance in session B than A in the move (but not control) group. However, this did not interact with language, suggesting it did not influence L1 or L2 lexical access specifically. Furthermore, picture-naming accuracy showed some changes across sessions (with poorer L1 performance in session B compared to A), but this was not modulated by group. Overall, therefore, there was no strong or consistent evidence for between-session changes specific to the move group that suggested a change in L1 or L2 lexical access after moving language environment (which was hypothesized based on the weaker links hypothesis).

The individual-difference analyses generally showed no relationship between changes in language production and (changes in) language profile. The exception was that participants in the move group who used their L1 less while living in the UK were more likely to show an increase in overall L1 picture-naming RTs between sessions. This suggests that at an individual level, lower L1 use can be associated with slower L1 retrieval. Some more general differences between the two groups were also observed, typically reflecting slightly better L2 performance in the move group. However, these differences were present even in the first session, suggesting that they were not a consequence of spending more time in the L2 environment. This aligns with some baseline differences found in terms of the move group’s higher use of L2-English before moving, potentially in preparation for their new academic degree in the UK.

We deliberately invited participants who had already been studying an English-related degree in China and who had reached an intermediate proficiency level before their move. Indeed, although we showed significant increases in L2 proficiency (and a decrease in self-rated L1 proficiency), these changes were small. In contrast, larger changes were observed for daily-life language use, in terms of both actual L1/L2 frequency of use and time spent in L1 and L2 single-language contexts. It is likely that larger changes in lexical access and overall L1/L2 performance are more easily observed in language learners who also show larger proficiency developments. Indeed, such changes were observed by Baus *et al.* [23] and Linck *et al.* [18], whose studies focused on people taking language classes abroad. Those language learners might show larger gains in language proficiency after moving to the L2 environment, which in turn could relate more strongly to potential changes in lexical access. It is possible that the process of language *learning* might also have larger consequences for language control than a change in language environment as such. When a certain proficiency has already been reached, moving to a new language environment does not appear to have a (large) impact on language control, as suggested by our study.

### 4.3. Limitations

Our study largely took place during the COVID-19 pandemic. This unfortunately negatively impacted the number of participants we could test as fewer international students moved to the UK. Our sample size was therefore smaller than intended, which resulted in lower power to detect between-session changes. However, power remained over 70%, and our Bayesian analyses further support our findings suggesting that no changes were present (although this evidence was only moderate). Although we were able to conduct the in-person parts of sessions A and B (with social distancing and face masks in the first wave), participants in the move group did have to complete their mandatory quarantine period before they could take part. This meant that within the first wave of testing in particular, participants were not tested immediately upon their arrival in the UK. While their language control might have started to adjust immediately (which could mask between-session changes), language control did not differ between the move and control group in either session, suggesting the quarantine period did not impact the overall results. Finally, it should be acknowledged that daily-life language experiences might have been different for international students during the pandemic. Less face-to-face interaction could have influenced the participants’ L2 language use while in the UK. It is possible longitudinal changes are observed when accompanied by larger increases in L2 use. However, our individual-difference analyses suggested no direct relationship between a bilingual’s language use and (changes in) language control. Furthermore, our participants did, as expected, show an increase in L2 use and time spent in L2 single-language contexts while in the UK, in addition to spending more time in dual-language contexts. Finally, participants in the two testing rounds (with the first one still having restrictions in place) did not show significant differences in their daily-life language experiences. This is likely also because even in the first testing round, restrictions concerned the use of face masks and avoiding large groups but did not impose lockdown-type restrictions that limited in-person contact.

## 5. Conclusion

In conclusion, our study shows that bilinguals use language control even when producing words in one language only in single-language contexts. This language control appears to be applied globally across the lexicon and in tasks varying in their demands on lexical selection. However, longitudinal analyses showed that language control does not necessarily adjust to a new language environment in which the L2 is used more frequently. This suggests that bilinguals are able to use their language control flexibly depending on the immediate context they are in, but that this language control might not change in response to the overall language environment a bilingual lives in.

## Data Availability

The pre-registration, data and analysis scripts can be found at OSF [40]. Supplementary material is available online [41].
